# Focal application of accelerated iTBS results in global changes in graph measures

**DOI:** 10.1002/hbm.24384

**Published:** 2018-10-01

**Authors:** Deborah C. W. Klooster, Suzanne L. Franklin, René M. H. Besseling, Jaap F. A. Jansen, Karen Caeyenberghs, Romain Duprat, Albert P. Aldenkamp, Anton J. A. de Louw, Paul A. J. M. Boon, Chris Baeken

**Affiliations:** ^1^ Department of Electrical Engineering, Eindhoven University of Technology Eindhoven the Netherlands; ^2^ Kempenhaeghe Academic Center for Epileptology Heeze the Netherlands; ^3^ Department of Neurology, Ghent University Hospital Ghent Belgium; ^4^ School for Mental Health and Neuroscience Maastricht University Medical Center Maastricht the Netherlands; ^5^ Department of Radiology Maastricht University Medical Center Maastricht the Netherlands; ^6^ Australian Catholic University Melbourne Australia; ^7^ University of Pennsylvania Pennsylvania Philadelphia; ^8^ Department of Neurology, Maastricht University Medical Center Maastricht the Netherlands; ^9^ University Hospital Brussels Jette Belgium; ^10^ Ghent University Ghent Experimental Psychiatry GHEP Lab Ghent Belgium

**Keywords:** accelerated intermittent theta burst stimulation, functional connectivity, graph analysis, transcranial magnetic stimulation

## Abstract

Graph analysis was used to study the effects of accelerated intermittent theta burst stimulation (aiTBS) on the brain's network topology in medication‐resistant depressed patients. Anatomical and resting‐state functional MRI (rs‐fMRI) was recorded at baseline and after sham and verum stimulation. Depression severity was assessed using the Hamilton Depression Rating Scale (HDRS). Using various graph measures, the different effects of sham and verum aiTBS were calculated. It was also investigated whether changes in graph measures were correlated to clinical responses. Furthermore, by correlating baseline graph measures with the changes in HDRS in terms of percentage, the potential of graph measures as biomarker was studied. Although no differences were observed between the effects of verum and sham stimulation on whole‐brain graph measures and changes in graph measures did not correlate with clinical response, the baseline values of clustering coefficient and global efficiency showed to be predictive of the clinical response to verum aiTBS. Nodal effects were found throughout the whole brain. The distribution of these effects could not be linked to the strength of the functional connectivity between the stimulation site and the node. This study showed that the effects of aiTBS on graph measures distribute beyond the actual stimulation site. However, additional research into the complex interactions between different areas in the brain is necessary to understand the effects of aiTBS in more detail.

## 1 INTRODUCTION

Transcranial magnetic stimulation (TMS) is a well‐established noninvasive neurostimulation technique used in a variety of experimental and clinical applications. A time‐varying current is sent through a coil placed tangential to the scalp. The magnetic field, induced by this time‐varying current, induces an electric field within the neural tissue in the brain, which is parallel to the current in the coil but has opposite direction. This electric field within the brain is able to modulate the activity of cortical neurons (Wagner, Valero‐Cabre, & Pascual‐Leone, 2007).

The effects of the repetitive application of TMS (rTMS) endure beyond the actual period of stimulation, affecting larger networks in the brain, which makes rTMS a potential treatment for various neuropsychiatric disorders (Klooster et al., 2016). The application of high‐frequency rTMS, delivering pulses at a frequency higher than 5 Hz, is currently FDA approved as treatment for patients with medication resistant major depressive disorder (MDD), which is approximately one‐third of all MDD patients. Left prefrontal high‐frequency rTMS has shown to be an effective and safe treatment in adult MDD patients documented as medication resistant (Pascual‐Leone, Rubio, Pallardó, & Catalá, 1996; George et al. 2010; George, Taylor, & Baron Short, 2013; Padberg & George, 2009; Baeken et al., 2013; Loo, McFarquhar, & Mitchell, 2008). The rationale to stimulate these parts of the cortex is based on earlier studies showing clear involvement of the prefrontal cortex (PFC) in the pathophysiology of MDD (Koenigs & Grafman, 2009). More specifically, the ventromedial PFC (VMPFC) shows hyperactivity, whereas the dorsolateral PFC (DLPFC) shows hypoactivity, as demonstrated by multiple imaging studies (Mulders, van Eijndhoven, Schene, Beckmann, & Tendolkar, 2015). Reversing these effects—decreasing the activity of the VMPFC or increasing the activity of the DLPFC—has been proposed as a possible mechanism by which rTMS treatment can achieve response and remission from depressive symptoms (George, 2010; Seminowicz et al., 2004).

Standard rTMS guidelines to treat depression follow mostly a daily pattern, with applied frequencies from 1 to 20 Hz, repeated for 4–6 weeks (Perera et al., 2016). With such protocols, clinical effectiveness remains however rather modest. To improve clinical outcome, new treatment parameters are currently under investigation. One new approach is *accelerated rTMS*, where a similar amount of stimulation sessions is concentrated over a couple of days instead of the more conventional daily sessions, spread over multiple weeks. Another line of research focuses on theta burst stimulation (TBS) (Huang, Edwards, Rounis, Bhatia, & Rothwell, 2005), where a particular set of parameter deliverables applies bursts of 3 stimuli at 50 Hz and is repeated every 200 ms (5 Hz, theta range). TBS has shown comparable clinical efficacy compared to rTMS but stimuli are delivered during a shorter period and usually with a lower intensity (Blumberger, Vila‐rodriguez, Knyahnytska, et al., 2018). Intermittent TBS (iTBS), the administration of 2 s of TBS alternated with 8 s rest, has been investigated for treatment of MDD (Bakker et al., 2015; Chistyakov, Rubicsek, Kaplan, Zaaroor, & Klein, 2010; Li et al., 2014), based on the excitatory character of the standard iTBS protocol (600 stimuli at 80% active motor threshold) (Huang et al., 2005).

To maximize clinical efficacy within a shorter time period, an intensive accelerated iTBS (aiTBS) protocol, consisting of multiple iTBS sessions per day, was recently tested as possible treatment for depression in our group. Duprat et al. (2016) showed a rapid significant decrease in depression severity symptoms assessed with the 17‐item Hamilton Depression Rating Scale (HDRS) (Hamilton, 1967) after 4 stimulation days. Although clinical effects were found both after sham and verum aiTBS, the most meaningful clinical outcomes regarding response and remission were observed 2 weeks after the aiTBS protocol, during follow‐up. While only 28% of the patients showed a 50% reduction of their initial HDRS score at the end of the stimulation procedure, response rates mounted up to 38% 2 weeks later, indicating delayed clinical effects. Furthermore, 30% of the responders were considered in clinical remission.

How aiTBS has the potential to improve depression symptoms over such a limited period in medication resistant MDD patients remains to be elucidated. Because it is known that the effects of stimulation are propagated through the brain via anatomical and functional connections (Amico et al., 2017; Fox et al., 2014), the effect of aiTBS might occur on a network level. In this study, the effect of this aiTBS protocol on the brain's network topology is investigated by means of graph analysis derived from resting‐state functional MRI (rs‐fMRI) data of a group of MDD patients. Graph analysis is a mathematical concept to quantify networks, for example, brain networks, according to various neurobiologically meaningful properties such as integration and segregation (Bortoletto, Veniero, Thut, & Miniussi, 2015; Rubinov & Sporns, 2010). Combining rs‐fMRI datasets before and after a brain stimulation protocol with graph analysis allows one to map the network changes throughout the whole brain induced by TMS, instead of just looking at single connections at a time, as it is done in many functional connectivity studies.

Previous studies have investigated the brain's network topology in patients with MDD. Graph analyses were performed based on cortical thickness (Mak, Colloby, Thomas, & O'Brien, 2016), voxel based morphometry measures (Lim, Jung, & Aizenstein, 2013), structural connectivity using diffusion MRI data (Ajilore et al., 2014; Chen et al., 2016; Korgaonkar, Fornito, Williams, & Grieve, 2014; Singh et al., 2013), or functional connections using rs‐fMRI datasets (Bohr et al., 2013; Li et al., 2016). The reported differences in graph measures between healthy volunteers and MDD patients were ambiguous. On one hand, some studies did not find differences, and on the other hand, increases in clustering coefficient, local efficiency, and path lengths were reported.

To study the effects of stimulation on network level, only few studies have been performed combining brain stimulation and graph theory: for example, Shafi et al. (2014) and Deng et al. (2015) used resting EEG data to examine the effects of continuous TBS and rTMS respectively. Shafi et al. (2014) showed frequency band dependent effects of stimulation on clustering coefficient and local efficiency: the beta band showed increases in clustering coefficient after cTBS, whereas alpha band showed decreases in clustering coefficient along with increased path length. Deng et al. (2015) showed reduced small‐worldness in the beta frequency band after stimulation. Vecchio et al. (2018) performed source localization on EEG data recorded before and after transcranial direct current stimulation (tDCS) and showed that anodal tDCS over the motor cortex reduces small‐worldness. Park et al. (2014), Polanía et al. (2011), and Cocchi et al. (2015) studied the effects of various stimulation techniques using task fMRI and rs‐fMRI data. Park observed a correlation between the motor performance change and the increase and decrease in global and local efficiency respectively, induced by 10 Hz rTMS (Park et al., 2014). Cocchi showed different effects of continuous versus inhibitory TBS represented by modularity, out‐degree participation index, and within‐module degree (Cocchi et al., 2015). Polanía et al. (2011) combined anodal tDCS over the motor cortex with rs‐fMRI derived graph measures and found increases in path length in the somatomotor areas after stimulation.

Besides the effect of aiTBS on graph measures, it will clinically be relevant to investigate if graph measures can be used as biomarkers to predict the outcome of this stimulation protocol. Previously, it has been shown that rs‐fMRI connectivities can be used for this purpose. Drysdale et al. (2016) derived four depression subtypes that seem to respond differently to rTMS treatment. And Fox et al. (2012, 2013) demonstrated that the clinical effects of rTMS are linked to the functional anti‐correlation between the subgenual anterior cingulate cortex (sgACC) and the stimulation spot in the left DLPFC. This anti‐correlation between the sgACC and parts of the left superior medial prefrontal cortex was also suggested to have predictive value for the outcome of accelerated rTMS in a cohort of MDD patients (Baeken et al., 2014), although in another accelerated iTBS this was not found to be that straightforward (Baeken, Duprat, Wu, De Raedt, & van Heeringen, 2017a). Nevertheless, Downar et al. (2014) showed in a cohort of MDD patients that the graph measure betweenness centrality can be used to distinguish responders from nonresponders to rTMS to the dorsomedial prefrontal cortex.

Specifically, this is the first study using graph analysis to investigate the clinical effects of the relatively new aiTBS treatment protocol. Graph analysis was performed on the whole‐brain level, using the clustering coefficient, global efficiency, small‐worldness, and modularity, and on the nodal level, using the degree, and the betweenness centrality as graph measures. Due to the presumably excitatory character of iTBS, we hypothesized that aiTBS would increase all four whole‐brain graph measures. On nodal level, we expected to find mostly increases in degree and betweenness centrality in nodes related to the pathophysiology of MDD. Furthermore, we expected that changes in graph measures would be linked to the clinical response. We also hypothesized that changes in functional connectivity, expressed by graph measures, would not only occur in the stimulated area (the left DLPFC), but will also be present in functionally connected regions.

## 2 METHODS

This study (http://clinicaltrials.gov/show/NCT01832805) was approved by the local Ghent University Hospital ethics committee and is in accordance with the declaration of Helsinki (2004). All patients gave written informed consent.

### 2.1 Inclusion criteria

Fifty right‐handed MDD patients were included in this study. MDD was diagnosed using the structured Mini‐International Neuropsychiatric Interview (MINI; Sheehan et al., 1998). All patients were at least stage I treatment resistant according to the Rush criteria (Rush, Thase, & Dube, 2003). They had a minimum of one unsuccessful treatment trial with selective serotonin reuptake inhibitors/serotonin and norepinephrine reuptake inhibitors (SSRI/SNRI). Medication was tapered off before the aiTBS treatment period, so all were medication‐free for at least 2 weeks before the start of the first stimulation session. More extensive information about the patients and clinical outcome can be found in Duprat et al. (2016).

### 2.2 Data acquisition

**Figure 1:**
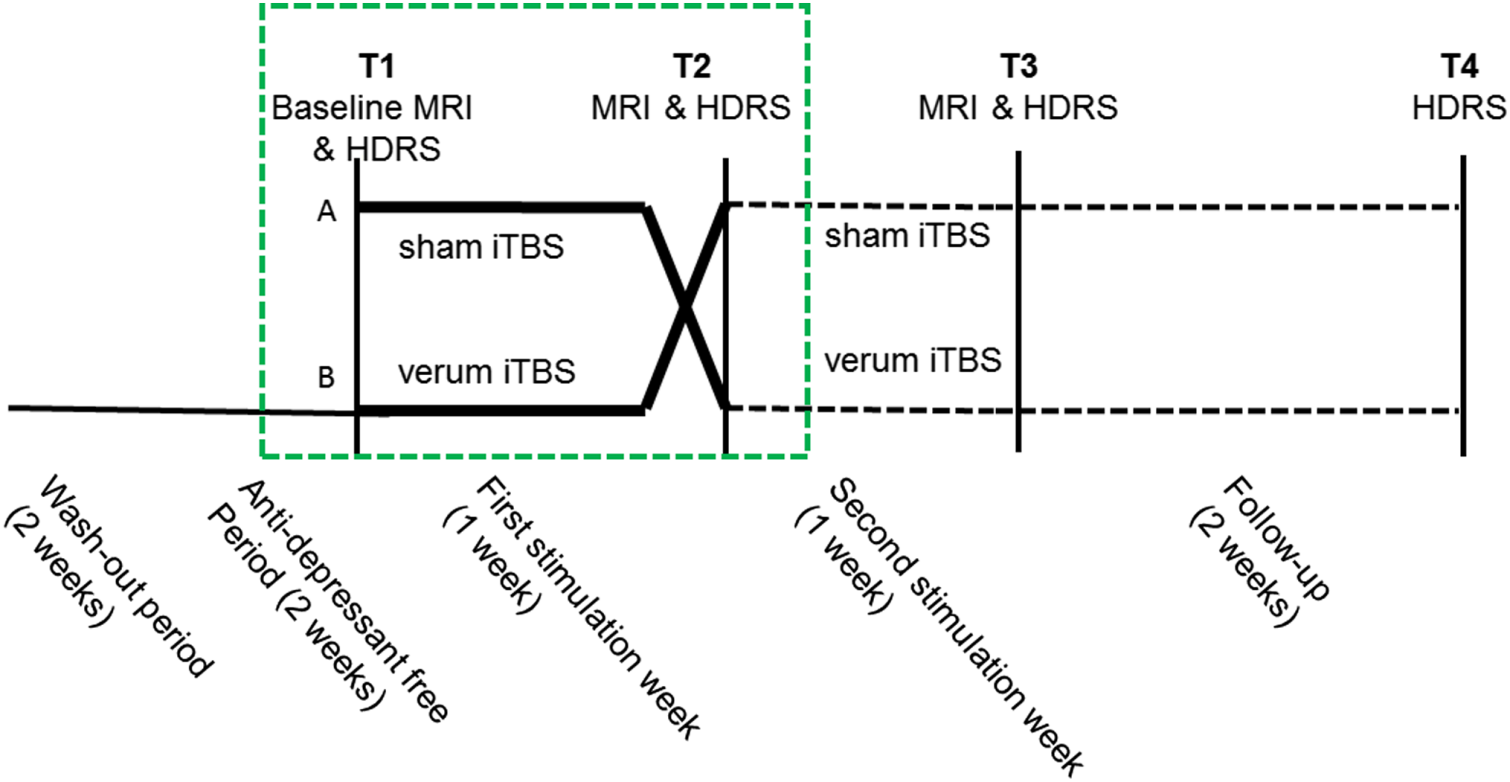
Design of the accelerated iTBS treatment procedure. After a washout period, all patients are at least 2 weeks anti‐depressant free before they are randomized to receive verum and sham accelerated iTBS treatment. Scheme adapted from Duprat et al. (2016) [Color figure can be viewed at http://wileyonlinelibrary.com]

The overall design of this randomized, sham‐controlled, double‐blinded, cross‐over trial is shown in Figure 1. Patients were randomized to receive first sham aiTBS followed by verum aiTBS (arm A in Figure 1) or the other way around (arm B in Figure 1). All patients first underwent baseline MRI (3 T Siemens TrioTim, Erlangen, Germany) on day 1 (T1) with anatomical imaging (MPRAGE, TR = 2,530 ms, TE = 2.58 ms, FA = 7°, FOV = 220 × 220 mm^2^, resolution = 0.9 × 0.9 × 0.9 mm^3^, 176 slices) and rs‐fMRI (EPI, TR = 2,000 ms, TE = 29 ms, FA = 90°, FOV = 192 × 192 mm^2^, resolution = 3 × 3 × 3 mm^3^, slice thickness/gap = 3/1 mm, 40 slices, 300 volumes, TA = 10.12 min). During the resting‐state measurement, patients were asked to stay awake with their eyes closed. On Days 2–5 and Days 9–12, verum or sham aiTBS was applied depending on the randomization order. A Magstim Rapid^2^ Plus^1^ magnetic stimulator (Magstim Company Limited, Wales, UK) connected to a verum or sham figure‐of‐eight shaped coil (Magstim 70 mm double air film [sham] coil) was used to apply the verum and sham stimulation respectively. On the 8th day (T2) and on the 15th day (T3), so 3 days after the stimulation, the imaging protocol was repeated. At the same days when imaging was performed (T1, T2, and T3) and additionally 2 weeks after the last stimulation (T4), depression severity symptoms were assessed using the 17‐item HDRS questionnaire (Hamilton, 1967).

Before the first stimulation session, the resting motor threshold (rMT) was determined based on motor evoked potentials (MEPs) induced in the right abductor pollicis brevis (APB) after applying single pulses to the hotspot. During four consecutive days, five daily sessions of iTBS were applied at 110% rMT to the left DLPFC: the center part of the midprefrontal gyrus (Brodmann area 9/46) based on structural MRI of each individual (Peleman et al., 2010). Positioning of the coil was maintained with the BrainSight neuronavigation system (Brainsight™, Rogue Resolutions, Inc). One iTBS session consisted of 54 trains of 10 bursts of 3 stimuli. Two seconds of stimulation were given in an 8 second cycling period. This adds up to 1,620 stimuli per session with a total number of 32,400 stimuli during the four‐day treatment. There were breaks of ~15 min between the stimulation sessions. During the stimulation, patients were blindfolded, wore earplugs, and were kept unaware of the type of stimulation (sham or verum) they received.

### 2.3 Graph analysis

Functional connectivity analyses were performed using the rs‐fMRI data from T1 and T2. In this first week of the study design, patients received either sham or verum aiTBS depending on the order of randomization. The second part of the study protocol, the period between T2 and T3 after the cross‐over, was not used to be able to study the pure effects of sham and verum aiTBS. The duration of the after‐effect of 4 days aiTBS is not yet known and as there was only a weekend between the stimulation weeks, effects of verum and sham might be crossed over into the second week.

**Figure A1:**
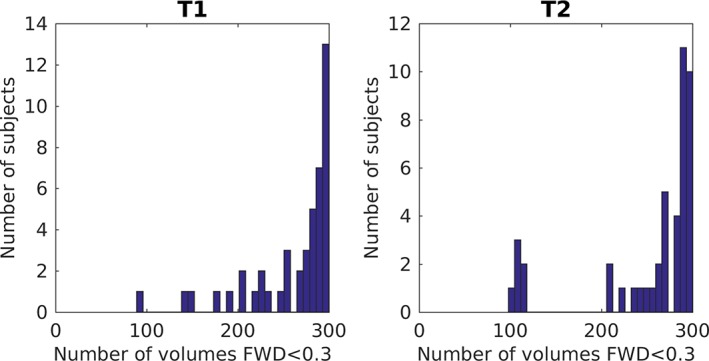
Number of volumes included in the analysis (FWD < 0.3) for every subject, per time‐point [Color figure can be viewed at http://wileyonlinelibrary.com]

Data were preprocessed with MATLAB 2015b (The Mathworks Inc., Natrick, MA) and SPM12 (Wellcome Trust Centre for Neuroimaging, London, UK) according to standard steps. After realignment, volumes with excessive motion, quantified as >0.3 mm framewise displacement, were discarded for further analysis. Complete datasets were excluded if more than 100 volumes had to be removed (see Appendix A, Figure A1). Six motion regressors, and additionally a white‐matter and cerebrospinal fluid regressor were used to correct the data using SPM's REST toolbox (Song et al., 2011). The latter two regressors were defined as the mean of the time‐series within the eroded white‐matter and cerebrospinal fluid masks, respectively. Temporal bandpass filtering was applied with cutoff frequencies of 0.1 and 0.01 Hz.

**Figure A2:**
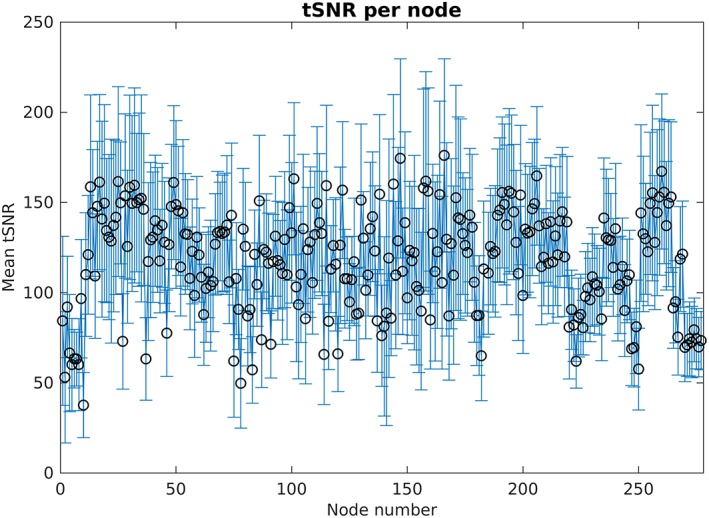
Overview of mean tSNR and standard deviation averaged over all subjects, per node. Nineteen nodes showed tSNR < 40 in more than 10% of the datasets and were excluded for further analysis (Table A2) [Color figure can be viewed at http://wileyonlinelibrary.com]

The brain datasets were parcellated using the parcellation scheme from Drysdale et al. (2016), using the 264 parcels, further referred to as nodes, from Power et al. (2011) and additionally 13 subcortical gray matter structures (see Appendix A, Table A1 for additional information). For all nodes, the mean time‐series was computed by averaging all the voxel time‐series belonging to that node. The temporal signal‐to‐noise ratio (tSNR) criterion was used to remove nodes with unreliable time‐series from further analyses. Nodes were discarded if tSNR <40 in more than 10% of the datasets (Liston et al., 2014). Furthermore, if more than 10% of the nodes within one dataset had tSNR <40, the dataset (both T1 and T2) were removed from further analysis (Appendix Figure A2).

For every patient and for both time points (T1 and T2), a connectivity matrix was calculated as the Pearson correlation between all the node time‐series, herewith rejecting the first 10 volumes to ensure scanner stability. The connections in this connectivity matrix are further referred to as edges. All edges are scaled to be in the range between zero and one (Schwarz & McGonigle, 2011) in a three‐step process. First, the range of the connectivity matrix was defined by subtracting the minimum value from the maximum value. Second, all edge‐values were divided by the range. Last, the minimum value of the new matrix was added which results in a scaled matrix between zero and one. This method was repeated for every subject and for every time‐point separately.

The MATLAB‐based Brain Connectivity Toolbox (Rubinov & Sporns, 2010) and the Graph Analysis Toolbox (Hosseini, Hadi, Hoeft, & Kesler, 2012) were used to calculate graph measures that quantify the brain's network organization (Bullmore & Sporns, 2009, 2012; Rubinov & Sporns, 2010). On whole‐brain level, four weighted graph parameters were calculated from every connectivity matrix: clustering coefficient, global efficiency, small‐worldness, and modularity. Here, high clustering coefficients are associated with high local efficiency regarding information transfer and robustness (Bullmore & Sporns, 2009). The modularity measure represents the way in which a network can be subdivided into modules: groups of nodes with a high number of within‐group links and a low number of between‐group connections (Girvan & Newman, 2002; Newman, 2004). Functional integration can be described by path length and efficiencies. High functional connectivity values can be translated to short path lengths and high efficiencies. The path length is the average of the shortest routes of information flow between pairs of nodes. Global efficiency can be calculated by inverting the path lengths. Moreover, the small‐worldness was calculated. Small‐world networks are assumed to be efficient, both locally and globally (Rubinov & Sporns, 2010). To calculate the small‐worldness, the clustering coefficient and path length were normalized by dividing them by their equivalents derived from random networks. Random networks were obtained using 20 randomization steps, leaving the degree of the connectivity matrix unchanged.

On the nodal level, two graph measures were calculated: the betweenness centrality and the degree. The betweenness centrality represents the fraction of shortest paths that pass through a certain node. Degree is a measure of interaction and can be calculated as the summation of all functional connections per node.

In general, graph measures are known to depend on the number of nodes and the average degree within a network (Wijk, Van, Stam, & Daffertshofer, 2010). Therefore, to obtain robust measures, every graph measure was calculated for a range of densities. The lowest density was set to 28% to prevent disconnected networks. The full density range comprises densities between 28 and 50% (in steps of 2%). Above 50%, connections are thought not be physiologically meaningful (Hosseini, Hoeft, & Kesler, 2012; Kaiser & Hilgetag, 2006). The area under the curve was calculated over this whole density range to obtain one robust, representative value for the graph measure per patient, per time‐point, and in case of the nodal analysis also per node.

### 2.4 Statistical analysis

In this study, functional connectivity, represented by various graph measures, was compared between T1 and T2 (Figure 1). Here, *ΔGM* is the change in graph measure (*GM*_*T*2_ − *GM*_*T*1_), and referred to as the effect size. Because of non‐normality of the graph parameters (see Appendix B), nonparametric permutation tests using 1,000 permutations were performed to investigate the difference between sham and verum stimulation on graph measures (ΔGM_sham_ vs ΔGM_verum_).

Significance level was set to *p* < .05 for the whole‐brain analysis. On the nodal level, additional multiple comparison correction was applied via the Holm–Bonferroni method, using the number of nodes for correction, but all findings with *p* < .05 were reported. Post‐hoc *t* tests were used to investigate the direction of the effects.

### 2.5 Spatial distribution

To study the assumption that the effect of aiTBS distributes via functional connections, the functional connectivity between the stimulation position in the left DLPFC and all the nodes showing an effect of verum stimulation over sham stimulation were calculated and correlated with the effect size. A circular region of interest (ROI), with a diameter of 1 cm, was positioned at the average stimulation position and a time‐series was derived by averaging all the time‐series of the gray‐matter voxels within the ROI.

### 2.6 Biomarker investigation

To investigate the predictive value of graph parameters on the clinical response to aiTBS, the baseline graph measures were correlated with the change in HDRS in terms of percentage (T2 with respect to T1 in the subgroup of patients receiving verum stimulation). Here, this means the lower the scores on HDRS changes in terms of percentage, the better the clinical response. Only significant correlations (*p* < .05) were reported.

## 3 RESULTS

**Figure 2:**
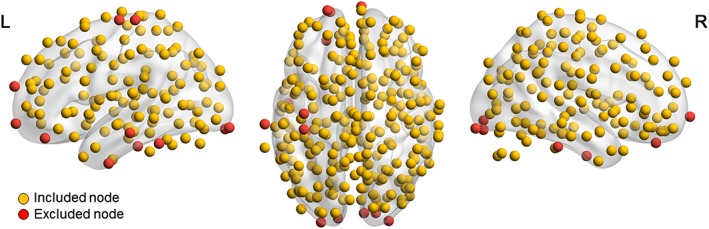
Overview of nodes used for graph analysis. After applying a tSNR criterion (at least 90% of the nodes should have tSNR >40), 19 nodes were excluded from the graph analysis (marked in red) [Color figure can be viewed at http://wileyonlinelibrary.com]

Given five drop‐out patients (due to a different diagnosis retrospectively, clinical improvement before the stimulation, or incomplete or wrongly timed MRI datasets), exclusion of seven patients (due to excessive motion in the MRI dataset at either T1 or T2), exclusion of three subjects based on the tSNR criterion, and three subjects did not have connected graphs within the density range, data from 32 patients were used for analysis. Of these patients, 14 received sham stimulation between T1 and T2 (arm A in Figure 1) and 18 received verum stimulation (arm B in Figure 1). Patient details and results on the clinical outcome of this stimulation protocol can be found in Duprat et al. (2016). Based on the tSNR criteria, 19 nodes (represented in red in Figure 2) were removed. Detailed information about the excluded nodes can be found in Appendix A, Table A2.

### 3.1 Whole‐brain network topology changes

On the whole‐brain level, stimulation caused a significant effect on clustering coefficient and global efficiency (*p* values <.01, <.01, .072, and .607 for clustering coefficient, global efficiency, modularity, and small‐worldness, respectively) (Appendix B). However, the effects did not differ between the subgroups receiving sham and verum stimulation. An overview can be found in Table 1. As can be seen in Table 2, changes in graph measures were not significantly correlated with changes in clinical outcome.

**Table 1:** Statistical overview of *p* values (permutation test with 1,000 permutations) representing the effect of stimulation type (verum vs sham) on whole‐brain graph measures

Graph measure	*p* value (tail = −1)	*p* value (tail = 0)	*p* value (tail = 1)
Clustering coefficient	.656	.688	.344
Global efficiency	.94	.12	.06
Modularity	.199	.378	.801
Small‐worldness	.528	.944	.472

**Table 2:** Correlation between the changes in whole‐brain graph measures versus the changes in clinical well‐being (after vs before stimulation)

	All subjects		Sham stimulated subjects		Verum stimulated subjects	
	Correlation coefficient	*p* value	Correlation coefficient	*p* value	Correlation coefficient	*p* value
Clustering coefficient	−0.21	.242	−0.35	.227	−0.20	.437
Global efficiency	−0.21	.254	−0.43	.125	−0.24	.344
Modularity	−0.07	.724	−0.03	.918	−0.03	.916
Small‐worldness	0.11	.551	0.21	.474	0.07	.796

### 3.2 Changes in nodal graph measures

**Figure 3:**
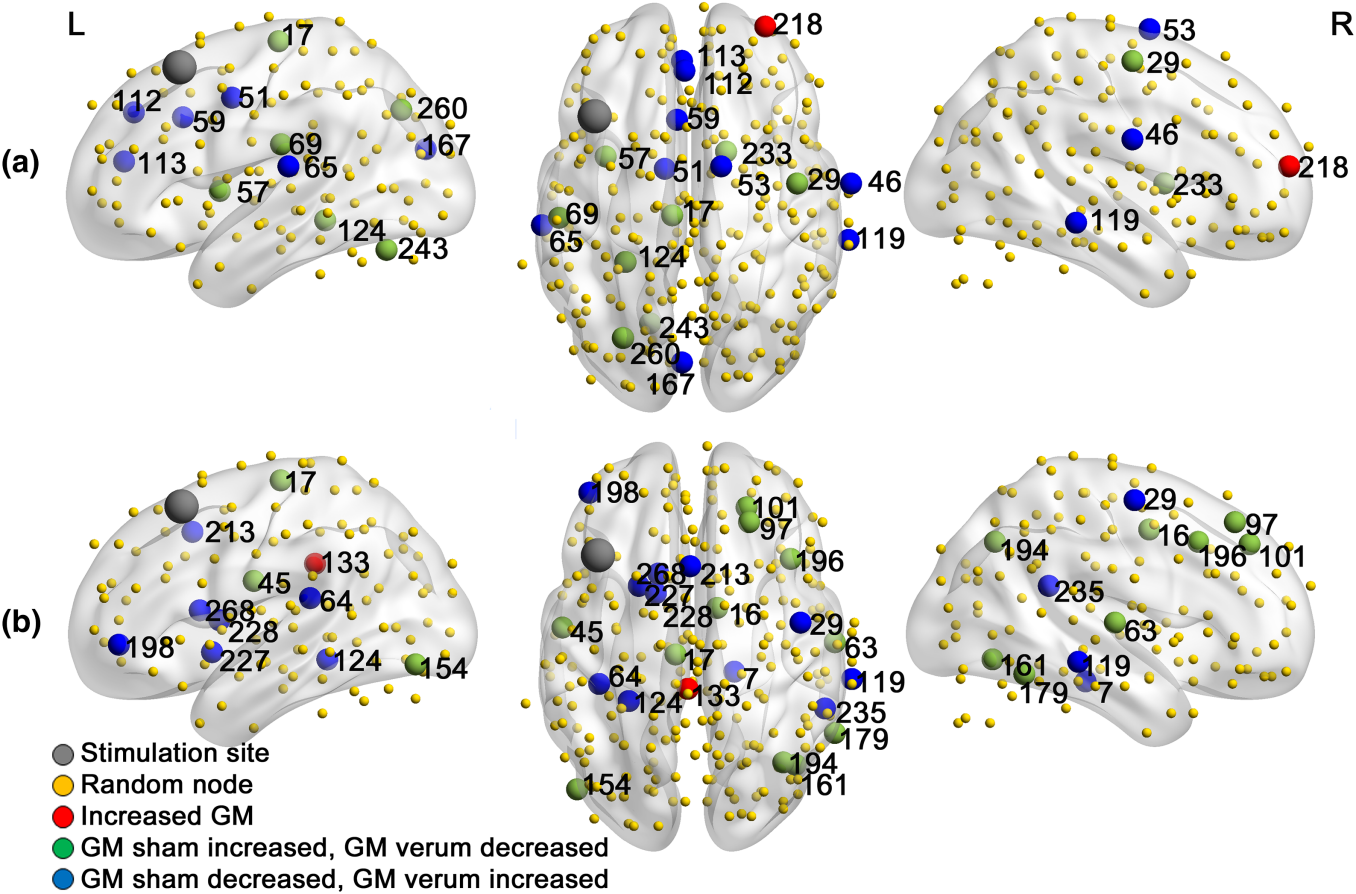
Overview of nodes showing significantly different effects of sham and verum aiTBS on (a) graph measures degree and (b) betweenness centrality [Color figure can be viewed at http://wileyonlinelibrary.com]

Figure 3 and Table 3 provide an overview of the nodes with significantly (*p* < .05) different effects of sham versus verum aiTBS. Only the betweenness centrality in the right supplementary motor area survived Bonferroni correction for multiple comparisons.

**Figure 4:**
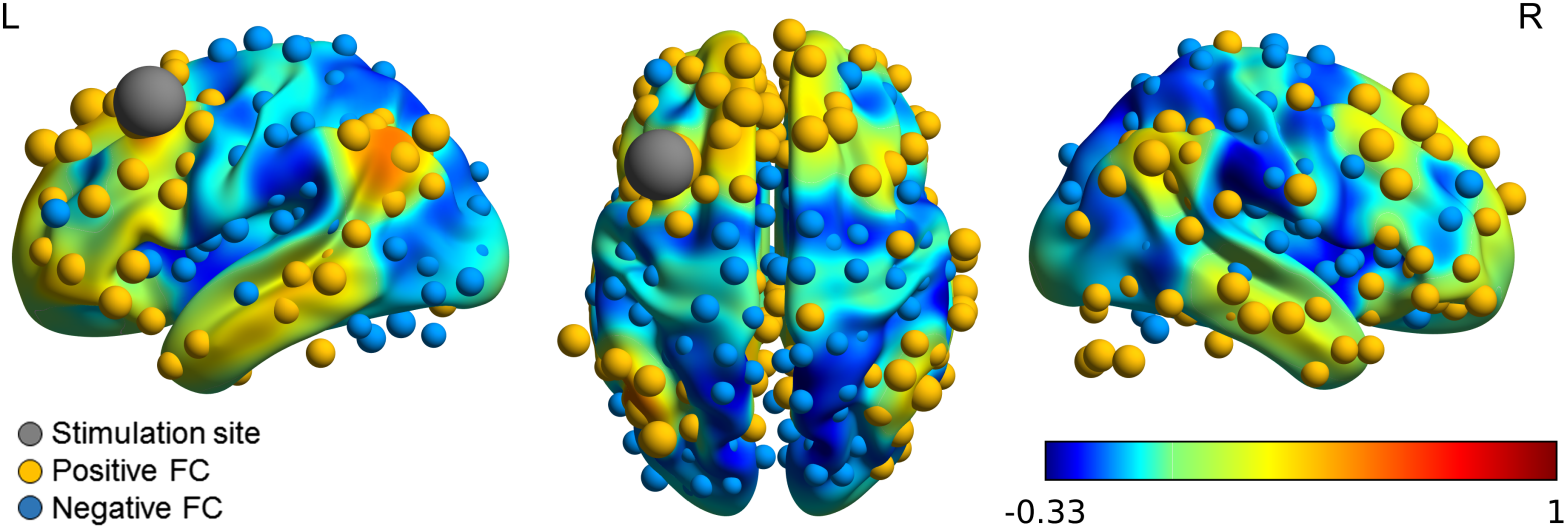
Functional connectivity (FC) with the stimulation area in the left DLPFC (MNI [−38, 20, 54]) as seed region. The volume shows the overall connectivity map obtained from http://www.neurosynth.org. Functional correlations with the nodes are shown in yellow and blue for positive and negative connections, respectively. The size of the nodes represents the strength of the connectivity [Color figure can be viewed at http://wileyonlinelibrary.com]

**Table 3:** Statistical overview of the node showing a significantly different effect between sham and verum aiTBS. Effects were defined as the change in graph measure (T2–T1)

Node number	Node name	*p* value	Correlation with stimulation site	Mean effect (sham patients)	Mean effect (verum patients)
Degree					
17	L paracentral lobule	.034	<0.01	1.921	−1.507
29	R precentral	.026	<0.01	1.677	−1.885
46	R postcentral	.027	0.07	−1.831	1.689
51	L cingulo‐opercular	.008	−0.07	−2.647	2.746
53	R supp motor area	.038	0.01	−2.099	1.802
57	L cingulo‐opercular	.02	−0.04	3.619	−0.609
59	L cingulo‐opercular (mid cingulum)	.016	0.01	−1.514	3.643
65	L supramarginal (auditory)	.049	−0.10	−1.873	1.389
69	L supramarginal (auditory)	.03	−0.07	0.950	−2.577
112	L frontal sup medial	.007	0.25	−1.851	2.121
113	L anterior cingulum	.049	0.12	−0.921	1.769
119	R mid temporal	.026	0.07	−3.112	0.448
124	L parahippocampal	.001	−0.07	2.534	−3.512
167	L cuneus	.031	−0.13	−0.886	2.639
218	R frontal middle	.039	0.02	0.365	3.851
233	R subcortical	.033	0.09	1.878	−2.227
243	L cerebellum	.033	−0.04	1.753	−2.101
260	L middle occipital	.048	−0.05	0.620	−2.632
Betweenness centrality					
7	R parahippocampal	.034	−0.03	−0.359	15.132
*16*	*R supp motor area*	0*	*−0.03*	*13.547*	*−22.387*
17	L paracentral lobule	.01	<0.01	10.903	−16.448
29	R precentral	.039	0.03	−3.283	8.738
45	L postcentral	.033	−0.04	6.856	−13.340
63	R temporal sup	.012	−0.10	4.909	−15.901
64	L rolandic oper	.009	−0.04	−22.929	1.287
97	R frontal sup	.003	0.16	10.439	−9.238
101	R frontal sup	.026	0.06	7.773	−6.713
119	R temporal mid	.003	0.07	−13.196	10.489
124	L parahippocampal	.025	−0.07	−17.820	2.300
133	L cingulum post	.028	0.12	15.210	2.517
154	L occipital inf	.021	−0.12	9.576	−7.326
161	R temporal inf	.004	−0.02	8.237	−12.659
179	R temporal inf	.029	‐ < 0.01	6.494	−5.580
194	R angular	.04	0.08	6.676	−7.374
196	R frontal mid	.045	0.16	9.377	−0.447
198	L frontal mid orb	.024	0.14	−11.100	4.519
213	L supp motor area	.015	−0.07	−14.444	3.072
227	L putamen	.023	0.03	−12.493	7.018
228	L subcortical	.011	0.08	−15.480	16.380
235	R temporal sup	.031	0.01	−11.220	3.228
268	L caudate	.004	0.05	−18.123	4.126

For all the nodes that showed a significantly different effect of sham versus verum stimulation, it was investigated if changes in graph measures were correlated with changes in clinical improvement. Table 4 shows an overview of the significant (*p* < .05) findings. A full overview can be found in Appendix C.

**Table 4:** Overview of nodes showing significant (*p* < .05) correlation between the changes in graph measures versus the changes in depression severity

Node number	Node name	All subjects		Sham stimulated subjects		Verum stimulated subjects	
		Correlation coefficient	*p* value	Correlation coefficient	*p* value	Correlation coefficient	*p* value
Degree							
124	L parahippocampal	−0.07	.69	−0.58	.03	−0.03	.90
Betweenness centrality							
45	L postcentral	0.60	<.01	0.69	.01	0.51	.03
213	L supp motor area	−0.33	.06	0.04	.90	−0.65	<.01

### 3.3 Propagation of effect via functional connections

The mean stimulation position within the left DLPFC within all 32 patients was [−38, 20, 54] (MNI coordinates in mm).

**Figure 5:**
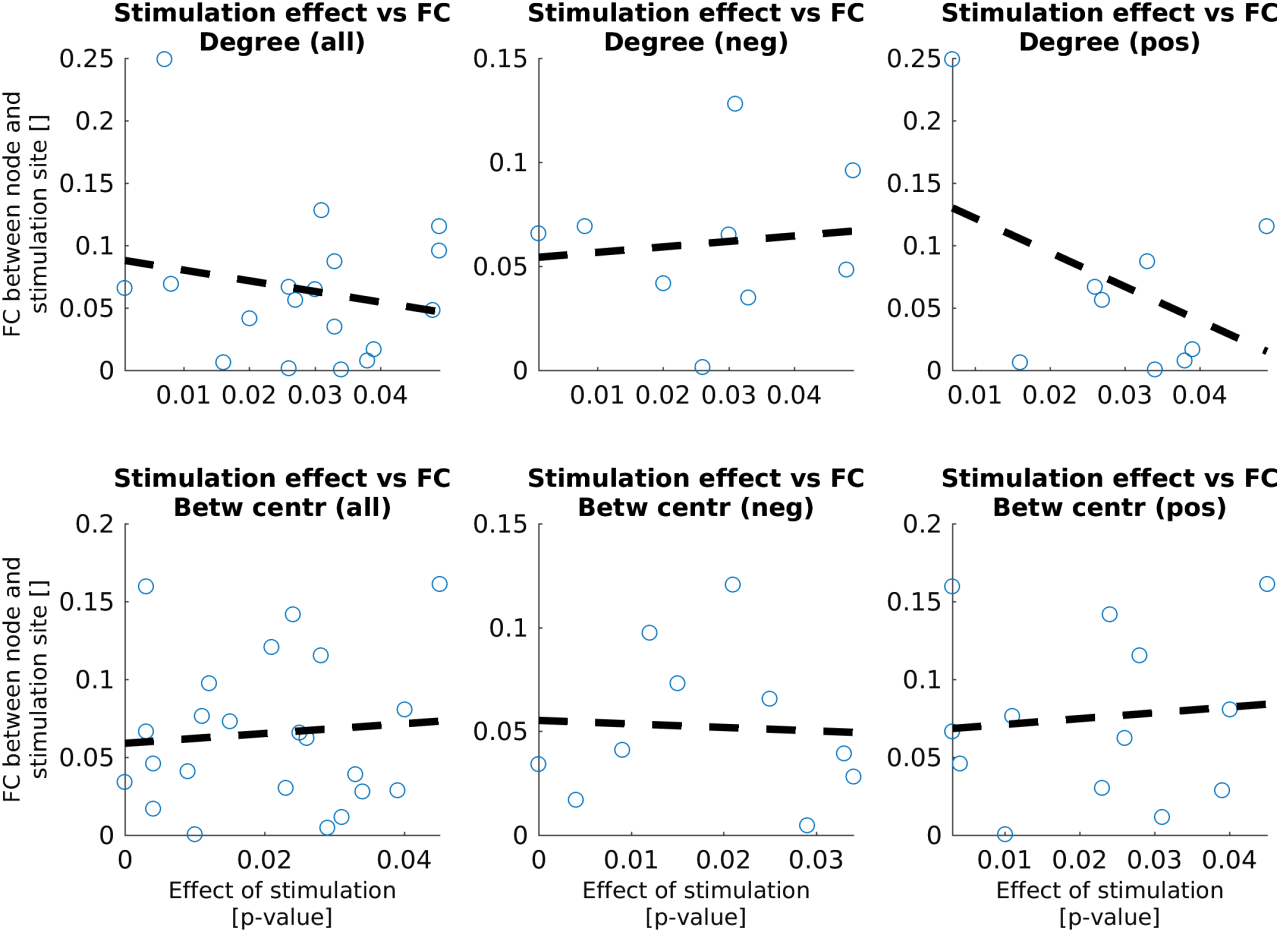
Correlation between the functional connectivities (FC) between the stimulation site in the left DLPFC and the nodes showing effects of verum stimulation with respect to sham stimulation and the strength of the effect. Statistical details can be found in Table 5 [Color figure can be viewed at http://wileyonlinelibrary.com]

For both graph measures that were calculated on nodal level, the functional connectivities between the stimulation site and the nodes with significant (*p* < .05) effect sizes were correlated with the *p* values. Figure 5 shows an overview of correlations, split into overall effects (absolute values of the functional connectivity) and negative and positive functional connections. Statistical details are summed in Table 5. No significant correlations were found. However, only based on findings within nine nodes, a large negative slope was found between the functional connectivity and the effect size of stimulation on the degree. This suggests that the effect of aiTBS on degree depends on the functional connectivity with the stimulation site: higher functional connectivities are linked to higher effect sizes (lower *p* values).

**Table 5:** Statistical details about the correlations between the functional connectivity and the effect size (belonging to Figure 5)

	Correlation coefficient	Slope	*p* value
Degree			
All	−0.20	−0.86	.42
Negative	0.12	0.26	.77
Positive	−0.44	−2.74	.24
Betweenness centrality			
All	0.09	0.32	.69
Negative	−0.06	−0.17	.88
Positive	0.10	0.37	.74

### 3.4 Potential of graph measures as biomarker

**Figure 6:**
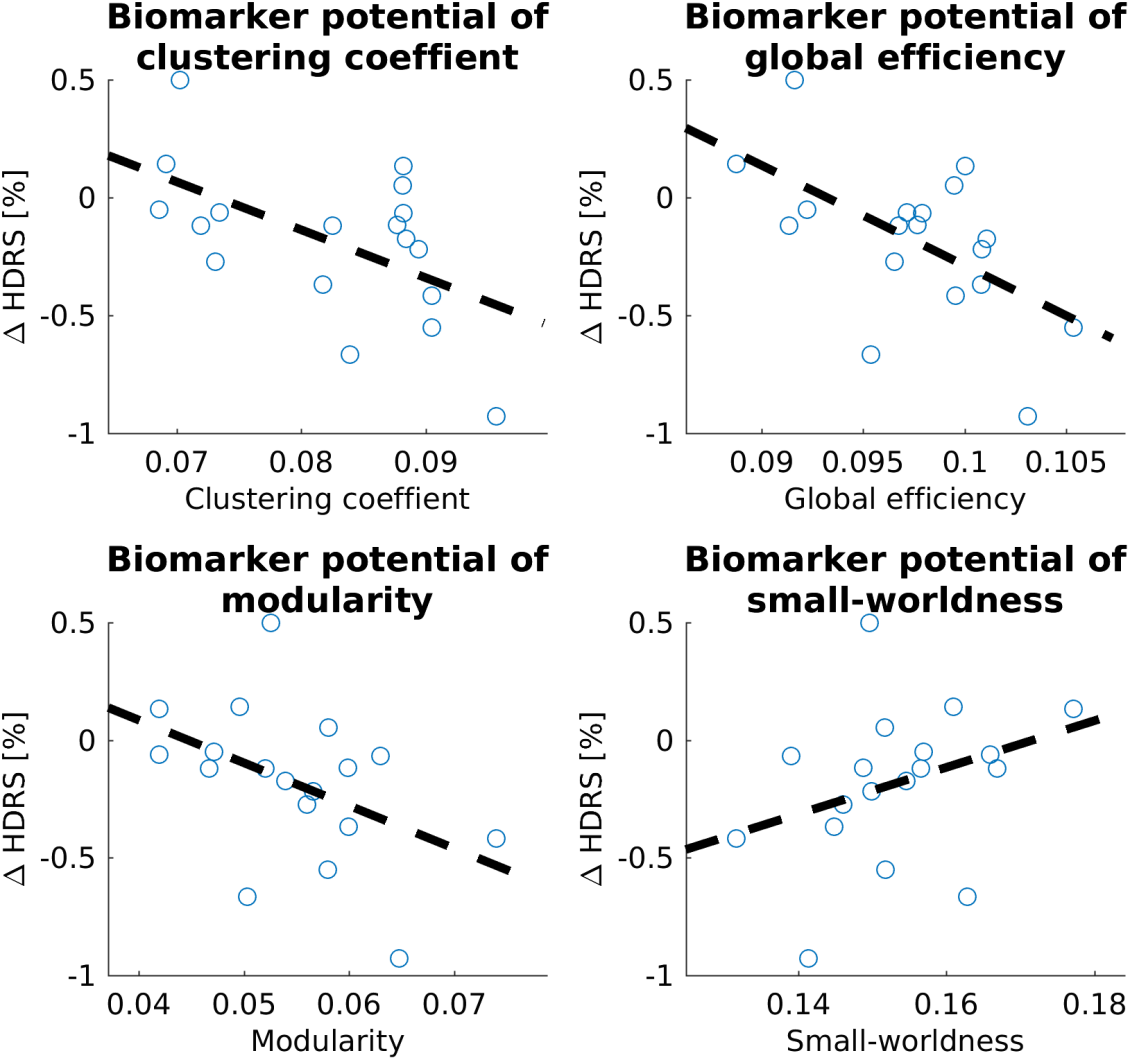
Potential of whole‐brain graph measures clustering coefficient, global efficiency, modularity, and small‐worldness to predict the percentage of clinical change of verum aiTBS. Statistical details can be found in Table 6 [Color figure can be viewed at http://wileyonlinelibrary.com]

Figure 6 shows an overview of the baseline whole‐brain graph measures versus the percentage of change in HDRS score, after versus before verum stimulation. Table 6 shows the statistical values.

**Table 6:** Statistical overview of the biomarker potential of the four whole‐brain graph measures

Graph measure	Correlation coefficient	Slope	*p* value
Clustering coefficient	−0.55	−20.3	.019
Global efficiency	−0.57	−42.5	.014
Modularity	−0.45	−18.18	.058
Small‐worldness	0.33	9.93	.173

Both the clustering coefficient and the global efficiency show a significant correlation between the baseline values and the changes in clinical well‐being. The negative correlation coefficient and slope indicate that higher baseline values may predict higher clinical effect of verum aiTBS.

**Figure 7:**
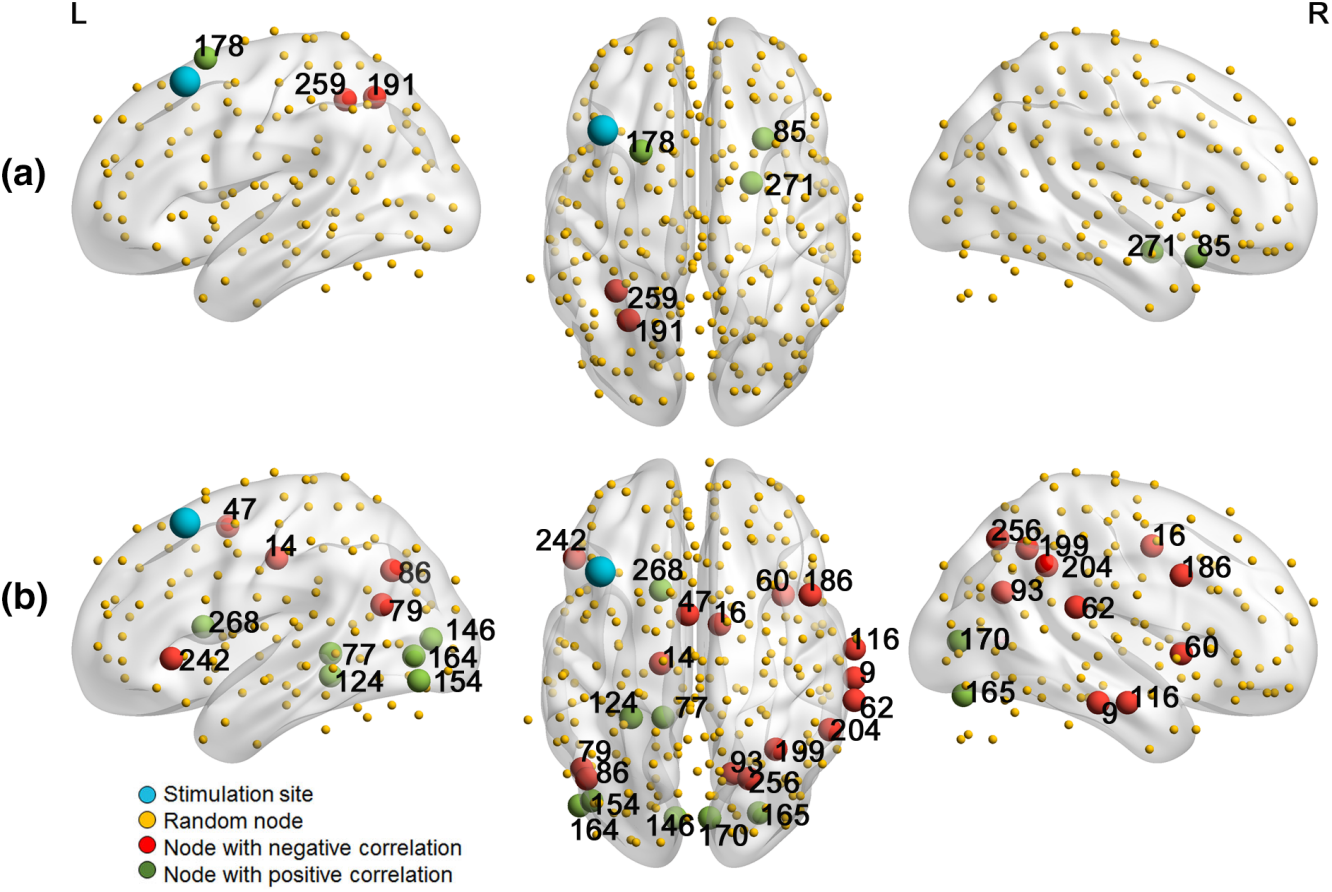
Nodes showing a significant (*p* < .05) potential of degree (a) or betweenness centrality (b) to predict the effect of verum aiTBS [Color figure can be viewed at http://wileyonlinelibrary.com]

A comparable analysis was performed on the nodal level, using the degree and the betweenness centrality as graph measures. Figure 7 shows an overview of the nodes showing significant (*p* < .05) effects and the belonging statistics can be found in Table 7.

**Table 7:** Statistical information about the nodes showing significant (*p* < .05) biomarker potential

Node number	Node name	Correlation coefficient	Slope	*p* value
Degree				
85	R insula	0.61	0.03	.007
178	L frontal superior	0.49	0.03	.038
191	L inferior parietal	−0.52	−0.03	.027
259	L inferior parietal	−0.53	−0.03	.025
271	R amygdala	0.53	0.03	.023
Betweenness centrality				
9	R middle temporal	−0.60	−0.01	.008
14	L medial somotomotor	−0.52	−0.01	.03
16	R supp motor area	−0.52	−0.01	.03
47	L supp motor area	−0.68	−0.01	.002
60	R cingulo‐opercular	−0.69	−0.01	.001
62	R superior temporal (auditory)	−0.62	−0.01	.006
77	L precuneus	0.56	0.01	.017
79	L midd temporal	−0.51	−0.01	.03
86	L angular	−0.55	−0.01	.018
93	R precuneus	−0.59	−0.01	.010
116	R middle temporal	−0.57	−0.01	.013
146	L Calcarine	0.60	0.01	.009
154	L occipital inferior	0.56	0.01	.015
164	L middle occipital	0.47	0.01	.049
165	R fusiform	0.52	0.02	.029
170	R Calcarine	0.54	0.01	.021
186	R frontoparietal	−0.56	−0.01	.017
199	R inferior parietal	−0.56	−0.01	.017
204	R supramarginal	−0.48	−0.01	.017
242	L frontal inferior	−0.49	−0.01	.039
256	R occipital superior	−0.52	−0.01	.028
268	L caudate nucleus	0.50	0.01	.036

## 4 DISCUSSION

This study aimed to use graph theoretical analysis to investigate the effects of the relatively new accelerated stimulation protocol to treat MDD patients, namely aiTBS, on the brain's network organization.

### 4.1 The effect of aiTBS on graph measures

#### 4.1.1 Whole‐brain results

On the whole‐brain level, no significant differences between the effects of verum stimulation versus sham stimulation were found, and changes in graph measures did not correlate with changes in depression severity symptoms. Previous studies (Ajilore et al., 2014; Lim et al., 2013) found no differences between graph measures clustering coefficients, path lengths, and small‐worldness in healthy subjects and patients with late‐life depression on whole‐brain level. Clinical effectiveness might not be linked to changes in whole‐brain graph measures. Even though aiTBS treatment in MDD patients does not influence the whole‐brain's network topology, it may have effects within subnetworks. Indeed, Tik et al. (2017) recently showed network‐specific increases in functional connectivity in one specific resting‐state network, containing the stimulated left DLPFC and the sgACC, after 10 Hz rTMS in a population of healthy subjects.

#### 4.1.2 Nodal results

On the nodal level, some nodes showed significantly different responses to verum and sham stimulation. Because these nodes are spread throughout the whole brain, this indicates that the effects of aiTBS are not restricted to the stimulation site. The nodes in proximity to the stimulated left DLPFC did not show differences between sham and verum responses. The direction of effects varied between nodes. Some nodes displayed significantly larger increases in graph measures after verum or sham stimulation and others showed increases after sham and decreases after verum or vice versa. Previously, it was demonstrated in similar types of MDD patients that clinical improvement after an accelerated high frequency rTMS paradigm was associated with significant increases of GABA (γ‐aminobutyric acid) concentrations in the stimulated area (the same left DLPFC spot that was also targeted here in this study) (Baeken, Lefaucheur, & Van Schuerbeek, 2017b). These GABA increases must be primarily considered as an “excitation” of GABAergic inhibitory neurons and pathways (Lefaucheur, Drouot, Ménard‐Lefaucheur, Keravel, & Nguyen, 2006). Both Kang et al. (2016) and Liston et al. (2014) have reported reductions in connectivity after 10 Hz rTMS, which is also assumed to have excitatory effects. However, one needs to keep in mind that according to Huang et al. (2005), the standard iTBS protocol is thought to result in excitatory effects. The aiTBS protocol is a modified form of the original iTBS protocol, not only in the number of pulses but also in the number of sessions. As it is known that modifications of stimulation protocols are able to reverse the polarity of the after‐effects (Gamboa et al., 2011; Gamboa, Antal, Moliadze, & Paulus, 2010; Gentner, Wankerl, Reinsberger, Zeller, & Classen, 2008; Murakami, Müller‐Dahlhaus, Lu, & Ziemann, 2012), it remains to be determined whether the net effects in the stimulated and connected areas are excitatory or inhibitory.

#### 4.1.3 Specific nodal effect

The most significant result (also the only finding that survived Bonferroni correction) was observed in the right supplementary motor area. Whereas the betweenness centrality increased after verum stimulation, it decreased after sham stimulation. This means that shortest paths between brain regions pass the right supplementary motor area. As TMS has been linked to changes in psychomotor performance before in the healthy as well as depressed state, this is of interest to explain to some extent the working mechanisms of this kind of stimulation. For instance, Baeken et al. (2010a) found improved psychomotor performance after high‐frequency rTMS treatment in medication resistant depressed patients. Also Hoeppner et al. (2010) showed a trend toward reduction of psychomotor agitation in MDD after high frequency rTMS. Our current findings indicate that left DLPFC aiTBS indeed may affect cortical areas involved in (psycho)motor actions.

In addition, more exploratory analyses revealed that the aiTBS treatment protocol shows effects on several (sub)cortical areas that can be linked to the pathophysiology of depression. For example, the effects of sham and verum aiTBS on degree differ in the left cingulo‐opercular nodes, which are part of the cingulo‐opercular network comprising the bilateral dorsal anterior cingulate cortices (dACC), the anterior insula, anterior prefrontal cortex, and the anterior thalamus (Sylvester et al., 2012). This network integrates visceral, autonomic, and sensory data to assess the homeostatic relevance or “salience” of internal and external stimuli, and the maintenance of tonic alertness or sustained attention (Sadaghiani & D'Esposito, 2015). The network also clears noisy information, suppresses distraction, and keeps cognitive faculties available for current processing demands (Sadaghiani & D'Esposito, 2015). Abnormalities in this network have been reported for obsessive compulsive disorder (OCD) (de Vries et al., 2017), psychosis (Sheffield et al., 2017), and mood and anxiety disorders (de Witte & Mueller, 2016). Of interest, Wu et al. (Wu et al., 2016) showed that depression symptom severity was significantly correlated with the connectivity values of this network. Indeed, increased activity in the dACC or insula during response conflict has been reported during negative mood states (Disner, Beevers, Haigh, & Beck, 2011).

Furthermore, several nodes that showed significantly different effects of verum and sham aiTBS, such as for example the parahippocampal nodes, nodes within the prefrontal cortex, and the posterior cingulum node, belong to the default mode network (DMN). The DMN is found to be activated during resting‐state functional imaging and de‐activated when performing cognitive tasks (Fox et al., 2005; Smith et al., 2009). When the brain is not engaged in externally driven cognitive processing, self‐referential processes are believed to predominate (Gusnard, Akbudak, Shulman, & Raichle, 2001). When clinically depressed, more activity in the DMN is observed (Disner et al., 2011). Changes in DMN activation have earlier been linked to anti‐depressant responses.

### 4.2 Spatial distribution of aiTBS effects

Previous studies have already shown distributed “network‐effects” of TMS (Fox et al., 2013; Fox et al., 2014). In this study, using nodes showing significantly different effects between verum and sham stimulation, the correlation between effect sizes and functional connectivity strengths did not reach significance. This indicates that the propagation of aiTBS‐effect from the stimulated area is not directly linked to the strength of the functional connections. Considering the network‐hypothesis, we hypothesize that the indirect effects of TMS occur at different levels. After the activation of brain areas connected to the stimulation site are activated, in the following steps, the brain areas connected to those areas are activated and so on. This could, at least partly, explain the occurrence of increases and decreases in graph measures in distinct areas of the brain.

### 4.3 Graph measures as biomarker

Clinical improvement was associated with higher baseline clustering coefficient or global efficiency on the whole‐brain level. This indicates that all nodes within the whole brain are better integrated. The effect of verum stimulation therefore seems to propagate more easily through the whole‐brain via functional connections, also to deeper structures involved in the deregulated neurocircuitry of depression.

On the nodal level, we found that graph measures in multiple nodes showed potential to predict the clinical effect. For example, a positive correlation between the baseline betweenness centrality and clinical effect was found in the left caudate nucleus. So lower betweenness centrality might be advantageous for clinical outcome. Given that the caudate has neural innervation from amongst others the prefrontal cortex, our left caudate nucleus findings could be linked to the application of left‐sided stimulation (Kang et al., 2016). Indeed, stronger connectivity between the dorsal prefrontal cortex and the (dorsal) caudate has been associated with depression severity (Furman, Paul Hamilton, & Gotlib, 2011; Kerestes et al., 2015). Furthermore, observations of increased connectivity with the DLPFC and the more ventral parts of the ACC in MDD was associated with heightened cognitive regulation of affect, usually problematic when clinically depressed; whereas reduced connectivity with the caudate results in worsening symptoms such as anhedonia, reduced motivation, and psychomotor dysfunction (Davey, Harrison, Yücel, & Allen, 2012). Of note, although the sgACC was not implicated in our findings, the structural and functional connections between the striatum (caudate) and the (sg)ACC are well known (Gabbay et al., 2013). In treatment‐resistant depression, the sgACC has been proposed as biomarker for response for a variety of interventions, including rTMS treatment (Fox et al., 2012; Fox et al., 2013; Weigand et al., 2017). However, for the latter application, the functional connectivity findings are not that straightforward (Baeken et al. 2017a; Baeken et al., 2014) and the aiTBS treatment delivered to the left DLPFC may have different neurobiological effects on the reward system (including the caudate), based on the level of anhedonia in the depressive state (Duprat, Wu, De Raedt, & Baeken, 2017). Indeed, it remains to be determined whether the left DLPFC is the best target to stimulate. Other prefrontal areas, such as the dorsomedial prefrontal cortex have been successfully stimulated in depressed patients (Downar et al., 2014), and alternatively when facing nonresponse, the right orbitofrontal cortex (OFC) was found to be an excellent alternative (Feffer et al., 2018). The right OFC is considered as a ‘non‐reward’ nexus (Cheng, Rolls, Qiu, Liu, & Tang, 2016) showing reduced functional connectivity in MDD patients. Together with our own findings on clinical improvement combined with baseline striatal (caudate) betweenness centrality, these observations suggest that left DLPFC aiTBS could be successful for a selected cohort of patients.

Furthermore, the degree in the right amygdala was significantly correlated with the clinical effects of verum aiTBS, suggesting that less connections to the right amygdala could be predictive for better clinical responses. Given that the amygdalae are involved in (in)effective emotion regulation in stress‐related disorders (Gold & Chrousos, 2002; Perlman et al., 2012) and in particular the right amygdala is implicated when processing negative information stressful events (Baeken et al., 2010b; Mothersill & Donohoe, 2016), it is of interest to note that increased baseline and sustained amygdala activity to antidepressant treatment is associated with clinical nonresponse in major depression (Fonseka, Macqueen, & Kennedy, 2018).

### 4.4 General limitations

This study has some general limitations that need to be considered. Notwithstanding that rs‐fMRI is a unique and powerful tool to investigate human brain organization, it is based on an inherently ambiguous measure reflecting dynamic couplings that are not yet fully understood. Interscan rs‐fMRI data have shown great variability. For example, Ning et al. (2017 1683) aimed to derive the optimal TMS stimulation position based on functional connectivity between the DLPFC and the sgACC and showed different results using resting‐state data from same subjects at different time‐points. Longer rs‐fMRI scans were suggested to reduce this variability. Moreover, various patient‐specific factors may also influence the outcome of a stimulation protocol (Silvanto & Pascual‐Leone, 2008). As referred to earlier, Drysdale et al. (2016) has shown that the sub‐type of depression could be related to the response to stimulation. Furthermore, the sustainability of the effects of aiTBS, or any type of stimulation treatment, are not yet exactly known. Pascual‐Leone et al. (1996) showed clinical responses in MDD patients for up to 6 weeks. Changes in functional connectivity are mostly reported on shorter time‐scales. EEG functional connectivity showed changes up to 70 min after rTMS (Thut & Pascual‐Leone, 2010). Also, changes might be specific over time, for example, Tik et al. (2017) only showed increased functional connectivity after 15 min, but not after 30 min, of rTMS. In this study, the effect of aiTBS was determined 3 days after the last stimulation session. Even though, aiTBS is a much more intense stimulation protocol compared to single day rTMS, changes in functional connectivity might have already faded out after 3 days.

## 5 CONCLUSION

This study showed that there are no differences between the effects of verum and sham stimulation on whole‐brain graph measures and that changes in graph measures are not correlated with clinical response. However, baseline values of clustering coefficient and global efficiency were found to have predictive value of the clinical response to verum aiTBS. On the nodal level, differences between sham and verum aiTBS were found throughout the whole brain, indicating that the effects of aiTBS distribute beyond the actual stimulation target. Knowledge about both functional connectivity changes and the potential use of graph measures as biomarkers could be important additions to novel neurostimulation protocols, as not only a better understanding on the underlying working mechanisms of aiTBS on the depressed brain may provide more insights, it may also guide future stimulation protocols to ameliorate treatment outcome.

## Figures and Tables

**Figure B1:**
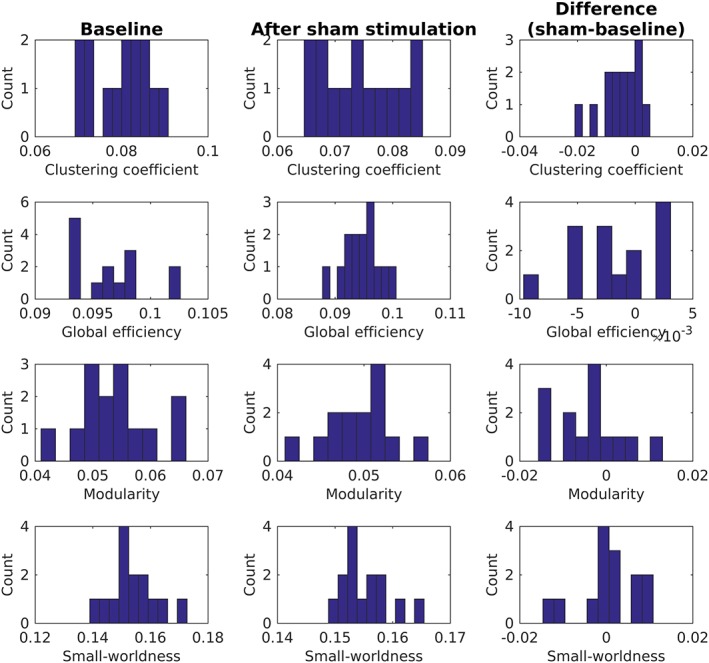
Overview of whole‐brain graph measures for subgroup of patients (*n* = 14) who received sham aiTBS during the first week of the stimulation protocol [Color figure can be viewed at http://wileyonlinelibrary.com]

**Figure B2:**
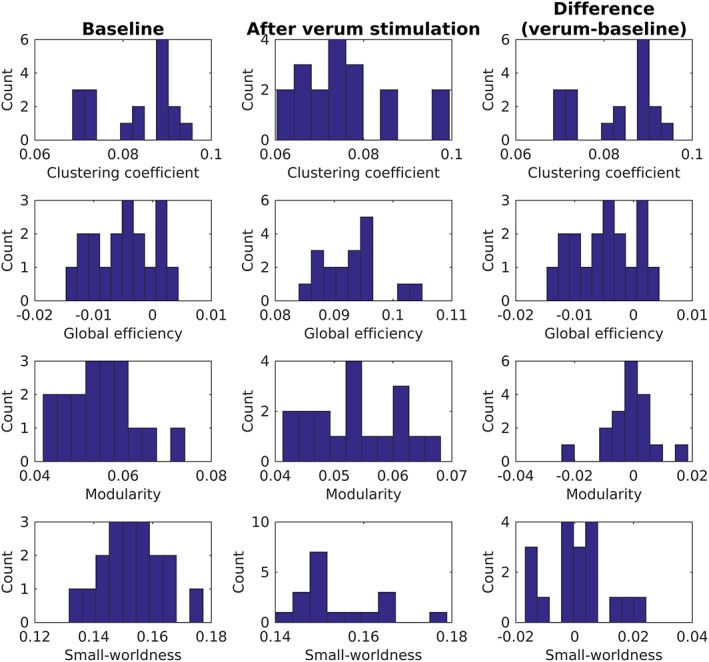
Overview of whole‐brain graph measures for subgroup of patients (*n* = 18) who received verum aiTBS during the first week of the stimulation protocol [Color figure can be viewed at http://wileyonlinelibrary.com]

## APPENDIX A: ADDITIONAL INFORMATION ON PREPROCESSING

### Motion parameters

Overview of the number of volumes per patient after removing the ones with framewise displacement >.3. Datasets with <200 volumes were discarded from further analysis. Note from Figure A1 that 5 datasets in T1 show excessive motion versus 6 in T2. With four overlapping datasets, this led to removal of 7 datasets for further analyses.

### Node selection

The first 264 nodes are resulting from the Power parcellation, as described in Power et al. (2011)). Thirteen additional nodes were appended, in accordance to Drysdale et al. (2016). An overview can be found in Table A1.

**Table A1:** Nodes that were added to the parcellation scheme proposed by Power et al. (2011))

Node number	*X* (MNI)	*Y* (MNI)	*Z* (MNI)	Node name
265	−9	10	−10	Nucleus accumbens (L)
266	10	10	−9	Nucleus accumbens (R)
267	1	25	−11	sgACC
268	−14	12	12	Caudate nucleus (L)
269	14	12	12	Caudate nucleus (R)
270	−20	−4	−15	Amygdala (L)
271	22	−2	−15	Amydala (R)
272	−28	−22	−12	Ventral hippocampus (L)
273	28	−22	−12	Ventral hippocampus (R)
274	−6	−38	−30	Locus coeruleus (L)
275	6	−36	−28	Locus coeruleus (R)
276	−4	−15	−9	VTA
277	0	−32	−24	Raphe nucleus

### tSNR

Figure A2 displays an overview of the mean tSNR (averaged over patients) per node. The nodes that were discarded for further analysis are listed in Table A2.

**Table A2:** Nodes that were removed from further analysis because the tSNR requirements were not reached

Node number	MNIx	MNIy	MNIz	Node name
1	−25	−98	−12	Left occipital interior
2	27	−97	−13	Right occipital inferior
4	−56	−45	−24	Left temporal inferior
5	8	41	−24	Right rectus
8	−37	−29	−26	Left fusiform
10	52	−34	−27	Right temporal inferior
27	−38	−27	69	Precentral left
37	−38	−15	69	Left somatosensory
75	6	67	−4	Right medial orbitofrontal
78	−18	63	−9	Left superior orbitofrontal
83	−68	−23	−16	Left middle temporal
114	−20	64	19	Left superior frontal
140	8	−91	−7	Right lingual
141	17	−91	−14	Right lingual
142	−12	−95	−13	Left lingual
182	−21	41	−20	Left middle orbitofrontal
247	33	−12	−34	Right temporal inferior
250	−50	−7	−39	Left temporal inferior

## APPENDIX B: GRAPH PARAMETER OVERVIEW

Figures B1 and B2 show the distributions of whole‐brain graph measures for the subgroup of patients receiving sham and verum aiTBS, respectively.

## APPENDIX C: FULL OVERVIEW OF CORRELATIONS BETWEEN CHANGES IN GRAPH MEASURES AND CHANGES IN CLINICAL WELL‐BEING IN NODES SHOWING SIGNIFICANT EFFECTS OF STIMULATION

A full overview of correlations between changes in graph measures and changes in clinical well‐being in nodes showing significant effects of stimulation can be found in Table C1.

**Table C1:** Full overview of correlations between changes in graph measures and changes in clinical well‐being in nodes showing significant effects of stimulation

Node number	All subjects		Sham stimulated subjects		Verum stimulated subjects	
	Correlation coefficient	*p* value	Correlation coefficient	*p* value	Correlation coefficient	*p* value
Degree						
17	−0.0452	0.8061	−0.0105	0.9717	−0.2375	0.3427
29	0.2749	0.1278	−0.118	0.6878	0.3886	0.111
46	−0.1538	0.4007	0.0406	0.8905	−0.1743	0.4891
51	−0.0663	0.7185	0.1236	0.6737	−0.0243	0.9237
53	0.2027	0.2659	0.2112	0.4686	0.3676	0.1334
57	0.098	0.5935	−0.1127	0.7013	0.0829	0.7437
59	0.1546	0.3982	0.308	0.2841	0.2623	0.2931
65	−0.011	0.9526	−0.2247	0.4399	0.3092	0.2119
69	−0.0686	0.7091	0.0415	0.8879	−0.3547	0.1487
112	0.0411	0.8235	0.0131	0.9645	0.2892	0.2445
113	0.1355	0.4595	0.224	0.4413	0.2288	0.3611
119	−0.2307	0.2039	0.152	0.604	−0.4557	0.0573
124	−0.0723	0.6943	−0.5791	0.03	−0.031	0.9028
167	−0.1504	0.4114	−0.3954	0.1617	0.0712	0.7788
218	−0.0583	0.7511	−0.034	0.908	0.0484	0.8489
233	0.3357	0.0603	0.3958	0.1613	0.2184	0.3839
243	−0.1695	0.3537	−0.3481	0.2227	−0.2205	0.3792
260	−0.084	0.6477	−0.2677	0.3547	−0.0759	0.7646
Betweenness centrality						
7	−0.255	0.1589	−0.0173	0.9532	−0.2897	0.2436
16	0.1441	0.4314	0.2126	0.4656	−0.1616	0.5217
17	−0.0247	0.8934	−0.024	0.9351	−0.2211	0.378
29	−0.1332	0.4674	−0.11	0.7081	−0.032	0.8997
45	0.596	0.0003	0.6874	0.0066	0.5075	0.0316
63	0.4115	0.0193	0.2543	0.3802	0.4287	0.0759
64	0.0122	0.9471	0.5075	0.064	−0.2301	0.3583
97	0.247	0.173	0.1566	0.5929	0.189	0.4525
101	0.3499	0.0497	0.3261	0.2551	0.2696	0.2794
119	−0.2289	0.2077	−0.4309	0.124	0.0293	0.908
124	0.1761	0.3349	0.2405	0.4076	0.3206	0.1946
133	−0.0752	0.6826	−0.1231	0.6751	−0.0.2093	0.4046
154	0.1378	0.452	−0.0042	0.9887	0.0901	0.7222
161	0.1588	0.3854	−0.25	0.3887	0.2791	0.2621
179	−0.0191	0.9175	−0.0158	0.9572	−0.1837	0.4655
196	0.1062	0.563	−0.0731	0.8039	0.1791	0.4771
198	−0.2706	0.1341	0.0539	0.8547	−0.3695	0.1313
213	−0.3315	0.0638	0.0391	0.8945	−0.6532	0.0033
227	−0.1596	0.3829	−0.1797	0.5388	0.0162	0.949
228	−0.279	0.122	−0.1476	0.6145	−0.2986	0.2287
235	−0.0431	0.8148	−0.0629	0.8307	0.1389	0.5825
268	−0.0992	0.5893	0.0465	0.8747	−0.0714	0.7784
